# Designing and Delivering Genomics Training for Community Health Workers: A Collaborative, Equity-Centered Approach

**DOI:** 10.21203/rs.3.rs-10780870/v1

**Published:** 2026-09-02

**Authors:** Caitlin G. Allen, Marie Smith, Vickie Reed, Ashley Evans, Tamika Williams, Sarah Covington-Kolb, Greg Green, Cassandra Wright Glenn, Brea Burke, Laurie Hoffma, Kelsey Jaeger, Charkarra Anderson-Lewis, Paula S. Ramos, Paige Menking, Jaslyn A. Grullon, Ingrid Wagner, Yue Guan, Audrey McCrary-Quarles, Susan Mayfield-Johnson

**Affiliations:** Wake Forest University School of Medicine; Prisma Health; The University of Southern Mississippi; South Carolina State University; Forsyth Technical Community College; University of South Carolina; University of South Carolina; Atrium Health Wake Forest Baptist; Tennessee Community Health Worker Association; Tennessee Community Health Worker Association; Wake Forest University School of Medicine; The University of Southern Mississippi; Emory University School of Medicine; Ponderosa Public Health Consulting; Wake Forest University School of Medicine; Wake Forest University School of Medicine; Emory University; South Carolina State University; The University of Southern Mississippi

**Keywords:** Community Health Workers, Genomics, Workforce Training, Health Equity, Community-Engaged Research

## Abstract

Equitable implementation of genomics and precision health initiatives require workforce models that support meaningful community engagement and improve access to genomic knowledge among historically underserved populations. Community health workers (CHWs) are trusted frontline public health professionals who are well-positioned to support genomics education, navigation, and research engagement, yet few genomic training programs have been developed specifically for CHWs.

The PREFER CHW (Partnering to Build Understanding of Genomics Responsibly) Training Program was developed through a collaborative, equity-centered approach involving academic institutions, CHW organizations, and community partners across the southeast United States. Program development incorporated participatory curriculum design, shared governance, train-the-trainer preparation, co-facilitate delivery models, and dissemination planning. Training content focused on foundational genomics concepts, family health history, risk and inheritance, ethics, and communication.

This community case study illustrates how collaborative implementation approaches can strengthen the relevance, acceptability, and sustainability of genomics workforce initiatives. Lessons learned may inform future efforts to support equitable integration of genomics into community and public health settings.

## Introduction

The potential of genomics to improve population health depends on equitable representation in genomic research and access to genomic services. Yet persistent inequities remain. For example, most genome-wide association study participants are of European ancestry,[1–4] despite decades of calls for diversity in genomics.[3] Structural and systemic barriers, including distrust rooted in historical harms,[5] concerns over data privacy,[6–8] lower access to health information,[9, 10] and failures in culturally resonant outreach [9–15] continue to limit participation from racial and ethnic minorities, rural residents, and people with lower socioeconomic status. These disparities limit not only those who benefit from genomics but also the generalizability and validity of genomic discoveries.[2]

The National Human Genome Research Institute (NHGRI) Strategic Vision emphasizes the need for a *diverse, interdisciplinary genomics workforce*, including entry-level professionals, to help ensure genomics benefits all communities. However, national efforts to diversify this workforce still focus disproportionately on individuals with college and graduate degrees. Community health workers (CHWs) are trusted, frontline health professionals who are well-positioned to bridge gaps,[16] yet there are limited training and infrastructure for CHW roles in genomics education, navigation, and research engagement.[17–19]

CHWs are uniquely positioned as trusted liaisons between communities and health systems. Studies show that CHW involvement improves recruitment, retention, and acceptability in research and clinical services, especially among populations often marginalized by biomedical institutions.[20, 21] CHWs also frequently become lasting community resources on topics introduced through training or project work. [22–24] Despite these strengths, CHWs remain largely absent from genomics and precision-medicine initiatives.[17, 18, 25] Evidence suggests CHWs are highly interested in genomics[18, 19] (68% in a national survey reported wanting more training),[19] yet most have no access to relevant training, career pathways, or role models. Without dedicated training, CHWs lack the grounding in genomics, ethical and social issues, data privacy, and clinical pathways needed to meaningfully support precision health. This limits not only their contributions to research but also opportunities for communities to access genomic knowledge in trusted, culturally sensitive ways.

Importantly, the challenge of preparing CHWs for genomic engagement is not solely a curricular problem. Traditional top-down training approaches that are developed without meaningful community participation risk reproducing the same power imbalances, mistrust, and misalignment that have historically limited equitable participation in genomics.[14, 15, 26] In this context, how training is designed, governed, delivered, and disseminated is just as consequential as what content is taught.

The PREFER CHW (Partnering to Build Understanding of Genomics Responsibly) Training Program was developed in response to these gaps, with the explicit goal of integrating community-engaged models into every phase of CHW genomics training. Rather than treating engagement as an adjunct strategy, PREFER CHW positions collaborative methods as core implementation tools that shape curriculum development, facilitation, governance, capacity building, and dissemination.

In this paper, we present PREFER CHW as a community case study that illustrates how community-engaged frameworks can be operationalized within a genomics training initiative for CHWs. We describe the rationale, context, and programmatic elements of the training, with particular attention to the collaborative methods used to support equity, relevance, and scalability. We highlight practical lessons for researchers, health systems, and community partners seeking to build equitable genomics workforce capacity.

## Context

### Setting

Wake Forest University School of Medicine is the primary site, with partnerships with community-based organizations and CHW networks that are expert CHW trainers. Partner sites include the Center for Community Health and Alignment at the University of South Carolina, North Carolina Community Health Worker Association, South Carolina State University, Southern Mississippi University, and the Tennessee Community Health Worker Association. The training was developed with funding through the NHGRI Entry-Level Modules (ELM) for Training the Genomics Research Workforce (R25) mechanism (Expired PAR-23–123: Entry-Level Modules (ELM) for Training the Genomics Research Workforce (R25-Clinical Trial Not Allowed), R25HG013479). The ELM program was created to support collaborations between a lead site and partner sites to develop new curricula to be implemented in programs that train entry level workforces.

### Details to Understand Key Programmatic Elements

We describe seven key program elements for the PREFER CHW training: curriculum development, trainer preparation, delivery support, implementation processes, evaluation metrics, dissemination, and individual and research context. Together, these elements operationalize community-engaged principles as key implementation strategies (Table 1).

### Curriculum Development

We developed the PREFER CHW curriculum through a participatory, equity-centered Delphi process that emphasized co-creation with community partners.[27–29] Core content areas for the curriculum included Genomics 101, family health history, risk and inheritance, and data privacy and ethics. A multi-phase Delphi process allowed us to iteratively refine curriculum content, incorporate feedback from CHWs, community members, and genomics experts to ensure accuracy, clarity, and relevance to the CHW role. Ultimately the curriculum we developed included real-world examples, plain language content, and alignment with CHW roles. Consistent with community-engaged frameworks, stakeholder engagement in curriculum design is essential to addressing structural barriers and ensuring training is responsive to community needs.[26] Participatory co-design and audience-tailored curriculum development have been shown to enhance relevance, uptake, and sustainability of interventions.[30] Thus, curriculum development was treated as an iterative process, which allowed for continuous refinement based on community input.

### Trainer Preparation

PREFER CHWs Trainers are CHWs with substantial experience training CHWs, but the specific genomics content they delivered was new. Trainer preparation followed a structured, staged model designed to support trainer knowledge about the PREFER CHW content, as well as practical application of the material. Trainers received access to all of the curriculum content. All trainers participated in two interactive, two-hour long workshops focused on skill-building, practice-based learning, and delivery of the curriculum. This approach allowed trainers to build confidence while integrating new knowledge into their existing experience as trainers. This approach to structured, staged learning formats has been shown to support training effectiveness and downstream dissemination in community-engaged initiatives.[31] Our focus was on preparing trainers not only to deliver genomics-specific content from PREFER CHW, but also to adapt materials for local contexts and audiences.

### Delivery and Supports

Training delivery was grounded in shared training leadership and adult learning principles. Sessions were co-facilitated by CHW leaders and genomics content experts, which reinforced the value of lived experience alongside technical expertise. Using shared leadership and co-teaching models have been shown to strengthen collaborative facilitation and improve participant engagement in training settings. [32] A comprehensive set of materials, which included slide decks, one-page conversation guides, frequently asked questions, and short educational videos supported delivery. To support professional recognition, CHWs received certificates of completion, continuing education credits where valuable, and documentation to support employer recognition and integration of training content into CHW scope of practice.

### Implementation Processes

Implementation was supported through structured governance, capacity-building strategies, and intentional equity-focused implementation strategies.[26] A multi-level governance structure for the training included an advisory board and CHW trainer group. These groups guided decision-making and ensured community representation through the design, delivery, and evaluation of the program. Structured governance and shared decision-making are associated with stronger outcomes in community-academic partnerships.[32, 33]

## Evaluation Metrics

The evaluation of PREFER CHW is guided by a mixed-methods approach. Quantitative data were collected through pre-course, post-course, and three-month follow-up surveys to assess changes in knowledge, self-efficacy, and retention of genomic concepts. Qualitative interviews are conducted to explore participant experiences and assess program outcomes across domains consistent with the RE-AIM implementation science framework.[34–38] This approach aligns with prior work emphasizing the importance of combining outcome evaluation with participant reflection and feedback to strengthen community-engaged interventions.[33]

## Dissemination

Dissemination has been included as an integral component of the PREFER training, rather than a downstream activity. Structured dissemination planning tools and curriculum have been shown to improve consistency and effectiveness of community-engaged dissemination efforts.[30] Additionally, designing for dissemination enhances likelihood of equitable reach and impact and supports trust and uptake among target populations.[39] Specific dissemination activities include the development of a public-facing website, which includes all course content and activities (e.g., workbook to accompany course). We also regularly share information with PREFER trainees via a listserv, including additional training opportunities aligned with PREFER, networking opportunities, and special topics sessions hosted by our team. The team has also actively disseminated information at national academic meetings (American Society of Human Genetics, American Public Health Association), and CHW-specific meetings across the southeast United States.

### Individual Researcher and Research Context

The PREFER CHW program explicitly incorporates attention to researcher positionality and the broader research context in which implementation occurred. Researchers engaged in ongoing reflection regarding their roles, assumptions, and influence within community–academic partnerships. Program design and implementation were informed by community leadership and local context, recognizing that successful engagement requires alignment with existing community priorities, infrastructure, and trust dynamics. Structured approaches to researcher–community partnership planning, including frameworks that emphasize shared goals and patient or community partnership, can strengthen implementation processes and outcomes. This emphasis on reflexivity and context provided a foundation for more equitable and effective collaboration between researchers and CHWs.[40]

## Discussion

The PREFER CHW Training program demonstrates the importance and feasibility of using community-engaged, equity-centered approaches to prepare CHWs to participate in genomics and precision health initiatives. We extend prior work on genomics education by illustrating not only what content may be appropriate for CHW genomics training, but also how collaborative implementation approaches can shape the relevance, acceptability, and potential sustainability of training programs. Across all phases of development and implementation of PREFER CHW, we positioned CHW and community engagement as a core component of the work, rather than as an adjunct activity.

Our experience suggests that CHWs are interested in and excited about engaging with genomic concepts, especially when training is scaffolded, grounded in plain language, and aligned with existing CHW competencies. Cohort-based learning, mentoring, trainer preparation, and peer support structures helped reinforce trainer and participant confidence and fostered networks that can support sustained engagement beyond the formal training period.[41] Notably, the training acknowledges the expertise CHW trainers and CHWs already bring, positioning CHWs as active contributors to genomics implementation, rather than passive recipients of technical knowledge. This is important, given the persistent concerns that genomics and precision-based approaches may be perceived as overly technical or disconnected from public health practice.[17, 20, 21, 42] By incorporating iterative co-design processes, shared facilitation models, and practical training tools, PREFER emphasized the application of genomic concepts within the context of trusted community relationships. These findings align with prior literature demonstrating that CHWs can serve as an effective intermediary between health systems and historically underserved communities, particularly when supported by culturally responsive training and infrastructure.[16]

We designed for equity from the earliest stages of implementation. Many genomics initiatives seek to improve representation or engagement after programs are already operationalized. In contrast, by design, PREFER CHW incorporated governance structures, compensation standards, dissemination planning, and community leadership into the foundational design of the program.[26, 32, 33] This approach reflects existing recommendations from community-engaged frameworks that emphasize the importance of power sharing, decision-making structures, and resource distribution for equitable implementation. Through early and ongoing engagement of CHWs and community partners, we were able to improve relevance, acceptability, and practical fit of the training content and materials to help avoid common mismatches between academic assumption and community needs.

Another important lesson from this program is the role of structured collaboration to support implementation. While community-academic partnerships are often discussed in terms of trust and relationship building, we included formal groups such as an advisory board, and CHW trainer leadership group that were critical to our success. These structures helped facilitate joint decision-making, clarified roles, and created mechanisms for ongoing adaptations and accountability. Prior work has shown similarly structured processes and partnerships can influence the effectiveness and sustainability of community-engaged initiatives.[26, 32, 33] In the context of genomics, where mistrust and concerns regarding exploitation remain substantial barriers to engagement, structured collaboration and governance may play an especially important role in supporting transparency and long-term collaboration.

Several limitations should be acknowledged. First, the PREFER training represents a single case study and may not fully reflect the needs, resources, or implementation contexts of all CHW programs or genomics initiatives. Second, evaluation activities are ongoing, and long-term impacts of the training have yet to be fully evaluated. Third, participating sites were organizations with existing expertise in CHW training and community engagement, which may limit transferability to settings with less established infrastructure. Additionally, because the program emphasized collaborative adaptations across sites, some implementation variability was expected and may influence outcomes across contexts.

In conclusion, the PREFER CHW program offers a practical example of how collaborative implementation approaches can be operationalized within genomics workforce development. As genomics and precision health initiatives continue to expand, there is increasing need for implementation models that prioritize trust, representation, accessibility, and community partnerships. CHWs represent an important workforce for supporting equitable engagement in genomics research and clinical care. Future work should examine long-term implementation outcomes, strategies for sustaining CHW genomics roles within health systems and community organizations, and the broader impact of CHW-led genomics engagement on participation, trust, and equitable access to genomics.

## Figures and Tables

**Table 1:** Key Program Elements of PREFER CHW Training

PREFER CHW Program Element	Details	Alignment with Community Framework
Curriculum Development	Core content is focused on Genomics 101, family health history, risk/inheritance, data privacy and ethics Delphi process for curriculum development	Stakeholder engagement emphasizes equity-centered co-creation to address structural barriers in curriculum design[26] Participatory co-design and audience-tailored curriculum development[30]
Trainer Preparation	**Format**: Webinar (orientation) → self-paced micro-modules → two 2-hour interactive workshops focusing on practice and local dissemination planning	Structured, staged learning formats guide training and dissemination[31]
Delivery and Supports	**Facilitation**: Co-taught by CHW leaders and genetic content experts. **Materials**: PowerPoint presentations, one-page conversation guides, FAQ scripts, short videos, and referral protocols; all written in plain language with bilingual options. **Recognition**: Certificates/CE credits; letters for employers; resume language for CHW scope of practice.	Adult learning approaches Shared leadership models (co-teaching CHWs + experts) enhance collaborative facilitation[32]
Implementation Processes	Governance/shared decision making with Advisory Board and CHW leadership group Capacity building: cohort mentoring Equity scaffolding: compensation standards for CHWs and community partners	Structured processes improve partnership outcomes[32, 33] Mentoring and peer networks strengthen partner capacity[41] Equity barriers: gaps in role clarity and compensation[43] CBPR approaches with shared decision making
Evaluation Metrics	Delivery of Pre-Course, Post-Course, and 3-month survey to assess program impact, self-efficacy, and knowledge retention. Conducting qualitative interviews to gain insight on Reach, Effectiveness, Adoption, Implementation, and Maintenance of program	Post-workshop survey evaluation of tools and team reflections[33] Supports RE-AIM aligned equity assessments[26]
Dissemination	Development of a dissemination toolkit with scripts, conversation guides, and short videos co-created with CHWs CHW-led dissemination planning to support site-specific outreach strategies Co-created, plain-language messaging tailored for community use	Structured planning templates guide consistent dissemination activities[31] Audience-tailored messaging, packaging, and distribution[30] Community engagement enables culturally tailored dissemination[39]
Individual Researcher and Research Context	Emphasis on researcher positionality and awareness of structural influences on implementation Researcher–CHW partnership planning informed by community leadership and program context	Provides a blueprint for structured researcher–community partnership planning[40]

## Data Availability

No datasets were generated or analyzed during the current study.
